# Beneficial Effects of Green Tea Catechins on Neurodegenerative Diseases

**DOI:** 10.3390/molecules23061297

**Published:** 2018-05-29

**Authors:** Monira Pervin, Keiko Unno, Tomokazu Ohishi, Hiroki Tanabe, Noriyuki Miyoshi, Yoriyuki Nakamura

**Affiliations:** 1 School of Food and Nutritional Sciences, University of Shizuoka, Suruga-ku, Shizuoka 422-8526, Japan; gp1747@u-shizuoka-ken.ac.jp (M.P.); miyoshin@u-shizuoka-ken.ac.jp (N.M.); yori.naka222@u-shizuoka-ken.ac.jp (Y.N.); 2 School of Pharmaceutical Sciences, University of Shizuoka, Suruga-ku, Shizuoka 422-8526, Japan; 3 Institute of Microbial Chemistry (BIKAKEN), Numazu, Shizuoka 410-0301, Japan; ohishit@bikaken.or.jp; 4 Department of Nutritional Sciences, Faculty of Health and Welfare Science, Nayoro City University, Nayoro-city, Hokkaido 096-8641, Japan; htanabe@nayoro.ac.jp

**Keywords:** green tea, catechin, EGCG, brain, cognitive function, Alzheimer’s disease, Parkinson’s disease, neuroprotection, epidemiology, inflammation

## Abstract

Tea is one of the most consumed beverages in the world. Green tea, black tea, and oolong tea are made from the same plant *Camellia sinensis* (L.) O. Kuntze. Among them, green tea has been the most extensively studied for beneficial effects on diseases including cancer, obesity, diabetes, and inflammatory and neurodegenerative diseases. Several human observational and intervention studies have found beneficial effects of tea consumption on neurodegenerative impairment, such as cognitive dysfunction and memory loss. These studies supported the basis of tea’s preventive effects of Parkinson’s disease, but few studies have revealed such effects on Alzheimer’s disease. In contrast, several human studies have not reported these favorable effects with regard to tea. This discrepancy may be due to incomplete adjustment of confounding factors, including the method of quantifying consumption, beverage temperature, cigarette smoking, alcohol consumption, and differences in genetic and environmental factors, such as race, sex, age, and lifestyle. Thus, more rigorous human studies are required to understand the neuroprotective effect of tea. A number of laboratory experiments demonstrated the benefits of green tea and green tea catechins (GTCs), such as epigallocatechin gallate (EGCG), and proposed action mechanisms. The targets of GTCs include the abnormal accumulation of fibrous proteins, such as Aβ and α-synuclein, inflammation, elevated expression of pro-apoptotic proteins, and oxidative stress, which are associated with neuronal cell dysfunction and death in the cerebral cortex. Computational molecular docking analysis revealed how EGCG can prevent the accumulation of fibrous proteins. These findings suggest that GTCs have the potential to be used in the prevention and treatment of neurodegenerative diseases and could be useful for the development of new drugs.

## 1. Introduction

Tea, a product of the plant *Camellia sinensis* (L.) O. Kuntze, is one of the most consumed beverages in the world. Green tea, black tea, and oolong tea are made from the same plant but are processed differently, depending on their degree of fermentation [1,2]. Among them, the health benefits of green tea have been most extensively studied, including its anti-cancer, anti-obesity, anti-diabetes, anti-inflammatory, and anti-neurodegenerative effects [1,2,3,4,5,6].

A cross-sectional study with 635 Japanese participants aged 69–71 examined the relationship between dietary patterns and cognitive function, and found that, among three dietary patterns—plant foods and fish, rice and miso (a fermented soy product) soup, and animal food—only plant foods and fish beneficially affected cognitive function. Since this dietary pattern is characterized by a high intake of green and other vegetables, soy products, seaweed, mushrooms, potatoes, fruit, fish, and green tea [7], green tea might have contributed considerably to that outcome.

Green tea contains a number of chemical compounds, including green tea catechins (GTCs), caffeine, and theanine, which may affect brain function. In a comprehensive review article, Chen et al. discussed the neuroprotective effects and mechanism of action of tea components, including tea catechins, theanine, caffeine, and theaflavins. They suggested that these bioactive tea components might be useful for neuronal degeneration treatment in the future [8].

**Figure 1:**
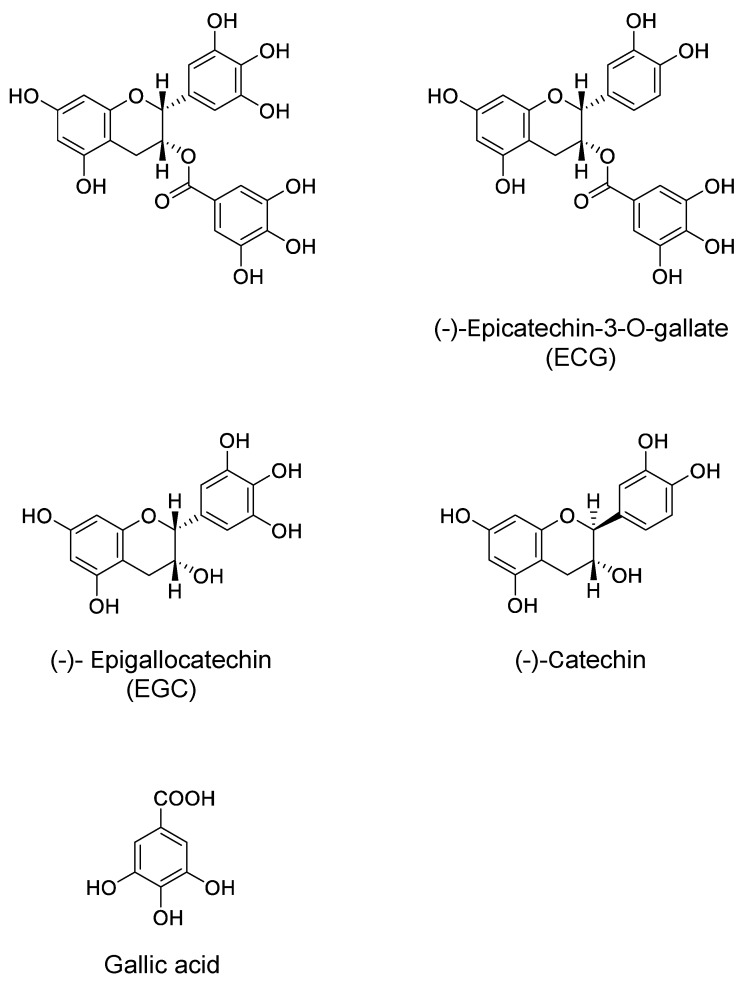
Chemical structure of epigallocatechin gallate (EGCG) and related compounds.

In this review, we update the findings about the neuroprotective activities of green tea and GTCs, especially (−)-epigallocatechin-3-*O*-gallate (EGCG) (Figure 1). We also discuss their mechanism of action, in which catechin-protein binding interaction is involved, based on the results obtained from computational molecular docking analysis (CMDA).

## 2. Observational Epidemiological Studies

### 2.1. Alzheimer’s Disease

Alzheimer’s disease (AD) is a neurodegenerative disorder with two major hallmarks, *β*-amyloid plague accumulation and neurofibrillary tangle formation. Some studies have reported that tea consumption is inversely associated with the risk of AD. For example, a cross-sectional survey of 2015 subjects aged 65 or older in Zhejiang province, Eastern China, found that the age-gender-standardized prevalence rates of dementia, AD, and vascular dementia were 13.0, 6.9, and 0.5%, respectively. Being elderly, low educational level, heavy smoking, heavy alcohol consumption, diabetes, and stroke were associated with dementia, whereas tea consumption was associated with a low prevalence of AD and severe cognitive impairment [9].

In contrast, several studies failed to show any beneficial effect of tea on AD, although some admitted tea’s beneficial effect on cognitive impairment. Kim et al. conducted a meta-analysis of 20 studies that included a total of 31,479 subjects and found that caffeine intake from coffee or tea was not associated with the risk of cognitive disorders. These authors listed five different studies in which tea consumption was not associated with the risk of AD [10]. For example, a 1992–1994 follow up study until 2001 of a population-based prospective study of 1836 Japanese Americans in King County, Washington, who were at base dementia-free, found that the hazard ratio (HR) for probable AD was 0.24 (95% confidence interval (CI), 0.09–0.61) in subjects who drank juice at least three times a week compared with those who drank juice less than once per week. The corresponding value is 0.84 (95% CI, 0.31–2.29) for those who drank juice one to two times per week. However, no association was observed for the dietary intake of vitamins E, C, or β-carotene, or tea consumption [11].

A study conducted afterward by Ma et al. evaluated the association between tea intake and the risk of cognitive disorders through a meta-analysis using the PubMed, Embase, and Wanfang databases. The overall pooled analysis of a total of 26 observational studies indicated that tea intake significantly reduced the risk of cognitive disorders (odds ratio (OR) = 0.65, 95% CI = 0.58–0.73). Subgroup analyses showed that tea consumption was inversely associated with the risk of cognitive impairment, mild cognitive impairment, cognitive decline, and ungrouped cognitive disorders. Their study suggested that tea’s beneficial effect on AD remains elusive, although daily tea consumption is associated with a decreased risk of cognitive decline in the elderly [12].

### 2.2. Parkinson’s Disease (PD)

Parkinson’s disease (PD) is the second most common neurodegenerative disease caused by the loss of nerve cells that produce dopamine. Several epidemiological studies indicated tea’s beneficial effect on PD. In 2009, Barranco Quintana et al. conducted a pooled analysis of 12 studies and found a clear protective effect of tea consumption in the risk estimate (OR: 0.83; 95% CI = 0.74–0.92) [13].

In a case-controlled study with 249 PD cases and 368 control subjects, the intake of coffee, black tea, and Japanese and Chinese teas was inversely associated with the risk of PD. The adjusted ORs compared with the highest to the lowest quartile were 0.52, 0.58, and 0.59, respectively (95% CI = 0.30–0.90, 0.35–0.97, and 0.35–0.995, respectively) [14].

In a prospective study that examined the relationship between habitual intake of dietary flavonoids and the risk of PD in 49,281 men and 80,336 women, Gao et al. observed that a greater intake of epicatechin (EC) and proanthocyanidin dimers was associated with a lower risk of PD. As a possible mechanism, the authors stated that dietary EC may stimulate the phosphorylation of the transcription factor cAMP-response element binding protein, a regulator of neuronal viability and synaptic plasticity, and inhibit NADPH oxidase activity. They also reported that proanthocyanidins may increase brain dopamine concentrations, inhibit monoamine oxidase-A activity, and reduce the 6-OHDA-induced dopaminergic loss [15]. Their study implied that catechin derivatives may be involved in tea’s beneficial effect on PD.

A meta-analysis of eight studies including 344,895 participants and seven studies with 492,724 participants assessed the association between tea and caffeine consumption on the risk of PD, showing a linear relationship. The smoking-adjusted risk of PD decreased by 26 and 17% when consumption was increased by two cups/day or 200 mg/day, respectively [16].

A case-controlled study of 75 patients with idiopathic PD and 75 control patients found that every additional glass of tea per day decreased the risk of PD by 0.8 times (OR = 0.8; 95% CI = 0.73–0.97, *p* = 0.02) [17].

These epidemiological findings support the beneficial effect of tea consumption, but some failed to provide clear evidence. For example, a cohort study conducted by Chen et al. on 74,941 women in urban Shanghai, aged 40–70, from 1996 to 2000, found that the risk of PD was inversely associated with exposure to second-hand tobacco smoke from husbands and tea drinking, and positively associated with education, although statistically insignificant. The age-adjusted OR was 0.8 (0.5–1.3) for tea-drinkers with continuous drinking of at least three times a week for six months or longer [18]. Thus, further studies are required to assess the association between tea consumption and the risk of PD.

### 2.3. Impairment in Global Cognitive Functions

Several observational epidemiological studies showed tea’s beneficial effects on cognitive function. Kim et al. [10] and Beydoun et al. [19] reviewed and listed those studies that had an effect. As summarized in Table 1, 7 out of 11 studies provided evidence for tea’s favorable effects. Some of these studies are described as follows. Kuriyama et al. reported the results of a cross-sectional study on green tea consumption and cognitive function [20]. The 1003 Japanese subjects aged 70 or older completed a self-administered questionnaire that included questions about the frequency of green tea consumption. The results indicated that a higher consumption of green tea was associated with a lower prevalence of cognitive impairment. After adjusting for potential confounders, ORs for cognitive impairment associated with different frequencies of green tea consumption were 1.00 (reference) for three or fewer cups per week, 0.62 (95% CI: 0.33, 1.19) for four to six cups per week or one cup per day, and 0.46 (95% CI: 0.30, 0.72) for two or more cups per day. Corresponding ORs were 1.00 (reference), 0.60 (95% CI: 0.35, 1.02), and 0.87 (95% CI: 0.55, 1.38) (*p* for trend = 0.33) for black or oolong tea, respectively, and 1.00 (reference), 1.16 (95% CI: 0.78, 1.73), and 1.03 (95% CI: 0.59, 1.80) (*p* for trend = 0.70) for coffee. That was the first large-scale epidemiological study to show that a higher consumption of green tea was associated with a lower prevalence of cognitive impairment in humans.

Similarly, a cross-sectional analysis of 2501 Chinese adults aged 55 or older and a longitudinal analysis of data from 1438 cognitively intact participants found that tea intake was significantly associated with a lower prevalence of cognitive impairment. Compared with the ORs for rare or no tea intake, the ORs for low, medium, and high levels of tea intake were 0.56 (95% CI: 0.40, 0.78), 0.45 (95% CI: 0.27, 0.72), and 0.37 (95% CI: 0.14, 0.98), respectively (*p* for trend <0.001). For cognitive decline, the corresponding ORs were 0.74 (95% CI: 0.54, 1.00), 0.78 (95% CI: 0.55, 1.11), and 0.57 (95% CI: 0.32, 1.03), respectively (*p* for trend = 0.042). These effects were most evident for black and oolong teas. Although an association of green tea with less cognitive impairment was found, the association was not meaningfully separated from that due to black or oolong tea consumption due to the small number of participants who only drank green tea [21].

A cross-sectional study of 681 Chinese nonagenarians and centenarians showed that, compared with subjects without cognitive impairment, male subjects with cognitive impairment had a significantly higher prevalence of two habits: smoking (*p* = 0.048 and 0.004, for former and current smokers, respectively) and alcohol consumption (*p* = 0.003 and 0.049, for former and current drinkers, respectively), but had a significantly lower prevalence for another two habits: tea consumption and current exercise. However, in female subjects, no association was found between cognitive impairment and these four habits [22].

A cross-sectional study involving 716 Chinese adults aged 55 or older in urban Singapore found that total tea consumption was independently associated with a better performance in global cognition, memory, executive function, and information processing speed. The protective effect of tea consumption on cognitive function was not limited to any particular type of tea (green tea, black tea, or oolong tea), whereas these effects were not demonstrated by coffee consumption [23].

Arab et al. also provided evidence of tea’s health benefits on brain disorders. Data on 4809 participants aged 65 or older from the Cardiovascular Health Study and with a median follow-up longer than 7.9 years indicated that participants who did not consume tea or coffee showed an annual decline in standard 3MS scores by an average of 1.30 points (women) and 1.11 points (men). Fully adjusted models showed modestly reduced rates of cognitive decline for some, but not all, levels of coffee and tea consumption for women, with no consistent effect for men. Caffeine consumption was also associated with reduced cognitive decline in women [24].

In a population-based prospective study with Japanese residents aged over 60, the incidence of dementia during a follow-up period of about 4.9 years was 5.3%, and that of mild cognitive impairment (MCI) was 13.1%. The multiple-adjusted ORs for the incidence of overall cognitive decline was 0.32 (95% CI: 0.16–0.64) among individuals who consumed green tea every day and 0.47 (95% CI: 0.25–0.86) among those who consumed green tea one to six days per week compared with those who did not consume green tea. The multiple-adjusted OR for the incidence of dementia was 0.26 (95% CI: 0.06–1.06) among individuals who consumed green tea every day compared with those who did not consume green tea. No association was found between coffee or black tea consumption and the incidence of dementia or MCI. These results indicate that green tea consumption is significantly associated with a reduced risk of cognitive decline [25].

A cross-sectional study of 1143 patients with a mean subject age of 68.9 in the Sado General Hospital, Niigata, Japan, found that the prevalence of cognitive impairment was 21.5%. Multivariate analysis revealed that age, low BMI (<21.1; OR 1.39; 95% CI: 1.12–1.72), a history of stroke, a history of myocardial infarction, low fruit consumption, and low green tea consumption were independently associated with a higher prevalence of cognitive impairment [26].

In an attempt to reveal possible associations between forkhead box, class O (FOXO) genotypes, and the effect of tea consumption on the cognitive disability of the elderly at advanced ages, Zeng et al. found that the interactions between carrying FOXO1A-266, FOXO3-310, or FOXO3-292 and tea drinking were significantly associated with a lower risk of cognitive disability. Experiments with animal and human cell models showed that the intake of tea compounds may activate FOXO gene expression, which in turn may positively affect cognitive function in the eldest population [27].

Thus, a considerable amount of evidence has indicated an inverse association of tea consumption with cognitive impairment, whereas several studies have failed to show such an association [20,21,22,23,25,28,29,30,31,32,33] (Table 1). Several examples showing no or even unfavorable association are discussed next. Mashal epidemiologically examined the effect of lifestyle factors on cognitive function in healthy aged subjects and found that tea intake may increase the risk of cognitive impairment [28].

In a one-year longitudinal study aimed at surveying a random sample of 1302 individuals 60 or older, the prevalence rate of amnestic MCI was 22.3%, and the rate of incidence was 96.9 per 1000 person-years. The prevalence rate of amnestic MCI in persons 70 or older was 30.3%, and the incidence rate was 145.6. Smoking, drinking tea, and surfing the Internet were not protective factors for this age group [34].

A Chinese longitudinal survey with 4749 cognitively intact adults aged 80 or older found that, compared with those who rarely or never consumed fruit, vegetables, meat, and soybean-derived products, participants consuming such products almost every day were 21, 25, 17, and 20% less likely to develop cognitive impairment, respectively. However, the consumption of fish, eggs, salt-preserved vegetables, tea, and garlic was not associated with cognitive impairment [35].

A meta-analysis of 20 epidemiological studies, which included 8398 subjects in six cross-sectional studies, 4601 in five case-control studies, and 19,918 in nine cohort studies, found that caffeine intake from coffee or tea was not associated with the risk of cognitive disorders [10].

A cross-sectional study among the Chinese elderly in which 9375 residents aged 60 or older were recruited, found that compared with non-consumption, the consumption of black tea showed a positive correlation with cognitive function after controlling for confounders (OR = 0.52; 95% CI: 0.28, 0.95), whereas green tea showed no significant difference (OR = 1.04; 95% CI: 0.72, 1.51) [36].

## 3. Human Intervention Studies

Several human intervention studies demonstrated the beneficial effects of green tea on brain functions, although others have not. Scholey et al. conducted a double-blind, placebo-controlled crossover study and found that 300 mg EGCG administration was associated with a significant overall increase in α-, β-, and θ-wave activities. EGCG consumption also increased self-rated calmness and reduced self-rated stress. These results suggest EGCG’s relaxing and refreshing properties, a beneficial effect of green tea intake [37].

In a randomized, placebo-controlled, single-blind study, 23 participants consumed one of the four test products: matcha tea, matcha tea bar (each containing 4 g matcha tea powder), placebo tea, or placebo bar. The results indicated that consumption of matcha, compared to the placebo, significantly improved tasks that measured basic attention abilities and psychomotor speed in response to stimuli, but the effect was barely present in other cognitive tasks [38].

Conversely, some studies failed to demonstrate that green tea or EGCG significantly affected cognitive functions, although some improvements were found in physiological biomarkers of brain function. In a double-blind, randomized controlled study conducted in Japan, participants were randomly allocated to the green tea or placebo group and consumed either 2 g/day of green tea powder (containing 220.2 mg of catechins) or placebo powder (containing 0.0 mg of catechins), respectively, for 12 months. The results for the 27 completed subjects showed that one year of green tea consumption did not significantly affect cognitive function. However, the level of malondialdehyde-modified low-density lipoprotein, a marker of oxidative stress, was significantly lower in the green tea group than the placebo group [39]. In a double-blind, placebo-controlled, crossover study, 27 healthy adults received placebo and two doses of EGCG. The consumption of 135 mg EGCG resulted in reduced cerebral blood flow in the frontal cortex in comparison with the placebo. However, 135 mg and 270 mg doses of EGCG caused no changes in cognitive performance or mood [40].

## 4. Laboratory Studies and Mechanism

### 4.1. Alzheimer’s Disease

The pathological hallmark of AD is the extracellular accumulation of amyloid plaques composed of fibrous amyloid. The proposed mechanisms for AD include microglia-triggered inflammation, over-activation of glutamate receptors, increased intracellular calcium levels, excessive generation of reactive oxygen species (ROS) and nitric oxide species, mitochondrial dysfunction, and synaptic dysfunction and loss [41]. Intracellular accumulation of the abnormal fibrous Aβ and phosphorylated tau proteins are key biomarkers in AD that are associated with inflammation [42], elevated expression of pro-apoptotic proteins [43,44], and oxidative stress [45], which lead to neuronal cell dysfunction and death in the cerebral cortex [46,47,48]. Hence, agents that suppress the formation of these biomarkers are thought to be useful for the prevention of AD [49].

In an AD model mice experiment using 3% d-galactose at a dose of 150 mg/kg body weight once daily for six weeks, or a dose of 2 mg/kg/day or 6 mg/kg/day of EGCG for four weeks, significantly reduced the accumulation of Aβ and released neuronal injury in the hippocampus of AD mode mice [50]. Similarly, in the TgCRND8 transgenic AD mouse model, which expresses multiple amyloid precursor protein (APP) mutations, oral administration of EGCG at 50 mg/kg/day for four months initiated at two months of age exerted beneficial effects on cognition and significantly reduced Aβ levels compared to untreated mice [51]. Furthermore, in another transgenic mouse model, APP/PS1 mice showed impaired spatial learning and memory in the Morris water maze test with the accumulation of Aβ. Oral administration of EC (50 mg/kg/day), combined with treadmill exercise for four months, protected against cognitive deficits and reduced Aβ levels [51]. In addition to the ability of GTCs to reduce Aβ, they also suppressed tau protein aggregation [51]. In an experiment using the APPSw transgenic AD mouse model, both intraperitoneal (i.p.) injection (20 mg/kg) for 60 days and orally treated EGCG (50 mg/kg) for six months regulated the tau protein profile and markedly suppressed the phosphorylated tau isoforms [52]. These studies demonstrated that green tea catechins inhibit Aβ and tau protein, exhibiting a potential to prevent and treat AD.

Down syndrome (DS) patients have an increased risk of AD neuropathology and AD-type dementia. Using Ts65Dn mice, a segmental trisomy model of DS that partially mimics DS/AD pathology, Catuara-Solarz et al. demonstrated that combined treatment of EGCG and environmental enrichment (EE) beneficially affected age-related cognitive impairment in Ts65Dn mice. The finding may be due to synergistic cellular and molecular effects between EE and EGCG, since they share common functions such as neuroplasticity enhancement, antioxidant activity, anti-inflammatory function, neuroprotection, promotion of the non-amyloidogenic proteolytic pathway of APP, and Dyrk1A kinase activity inhibition. Thus, a combination of EE and EGCG may provide a viable therapeutic approach to ameliorate age-related cognitive decline in DS [53].

Pathogenic Aβ formation is inhibited by α-secretase, which cleaves the Aβ domain of APP to generate soluble APP-α (sAPP-α), and EGCG was shown to increase sAPP-α in AD animal models. Based on the epidemiological studies demonstrating the association between fish oil consumption and reduced dementia risk, Giunta et al. examined whether co-treatment with fish oil and EGCG would reduce AD-like pathology in Tg2657 mice [54]. Co-treatment of N2a cells with fish oil and EGCG enhanced sAPP-α production compared to either compound alone. Bioavailability of EGCG was enhanced by fish oil co-supplementation. Fish oil and EGCG showed a synergetic effect on inhibition of cerebral Aβ deposits, suggesting that co-supplementation has therapeutic potential for the treatment of AD.

### 4.2. Parkinson’s Disease

PD is characterized by the presence of Lewy bodies mainly composed of ubiquinated α-synuclein, neurofilaments, synaptic vesicle protein, and parkin. Lewy bodies can be involved in disorders such as the release of free radicals, excessive generation of nitric oxide species, enhanced c-Jun N-terminal kinase pathway activated-apoptosis, microglia-triggered inflammation, and disruption of protein degradation pathways [41]. Hence, interference with these events would help prevent neurodegenerative diseases. A number of cell-based and animal experiments have provided evidence to support that tea and GTCs have beneficial effects on brain disorders.

In an PD model mice experiment using *N*-methyl-4-phenyl-1,2,3,6-tetrahydropyridine at a dose of 24 mg/kg (i.p.) for four days, pretreatment of mice with either green tea extract (0.5 and 1 mg/kg/day; i.p.) significantly protected against drug-induced dopamine decrease. In addition, oral administration of EGCG (2 and 10 mg/kg) also prevented the decrease of dopamine induced by the drug [55]. In another study using the same mouse model, administration of EGCG at two different doses (10 mg/kg and 50 mg/kg) reduced the drug-induced neuronal cell death rate to less than 50% [56].

In the human α-synuclein transgenic drosophila model of PD, the flies exhibit locomotor dysfunction as they age. An epicatechin gallate supplemented diet (final concentrations: 0.25, 0.50, and 1.0 μg/mL) that was fed for 24 days showed a significant dose-dependent delay in the loss of climbing ability, and reduced oxidative stress and apoptosis in brain cells [57]. Additionally, in an in vitro study using PC12 cells, 200 μM EGCG or ECG exhibited significant inhibitory effects against 6-hydroxydopamine-induced oxidative stress and apoptosis [57].

Intrastriatal injection of 1-methyl-4-phenylpyridinium (MPP) in rats resulted in the overproduction of free radicals, leading to the production of oxidative stress, decreased levels of dopamine, and behavioral disorders. Oral administration of 100 mg/kg EC before MPP injection significantly prevented dopamine decay and reduced circling behavior related to the damage induced by MPP [58].

In an experiment using 1-methyl-4-phenyl-1,2,3,6-tetrahydropyridine (MPTP)-induced PD mouse model, Zhou et al. found that EGCG treatment restored the movement behavior of the mice impaired by MPTP and prevented MPTP toxicity in tyrosine hydroxylase-positive cells in the substantia nigra pars compacta region [59]. Additionally, EGCG reduced the serum levels of inflammatory factors TNFα and IL6. These findings indicate that EGCG exerts neuroprotective effects in the PD mice model possibly through modulating peripheral immune response.

These studies suggest the potential use of green tea polyphenol as a therapeutic agent for the prevention and treatment of PD.

### 4.3. Other Neurological Impairments

In a comprehensive review, Singh et al. summarized the cellular and animal studies on the neuroprotective effects of EGCG and GTCs [36]. EGCG and GTCs may exert their effects by mechanisms including activity as an antioxidant (six studies), modulation of signaling pathways (six studies), inhibition of protein aggregation (eight studies), and modulation of apoptosis (six studies). For example, in a study conducted by Kang et al., EGCG inhibited catecholamine-*O*-methyltransferase-mediated *O*-methylation of L-dihydroxyphenylalanine (DOPA) with an average IC_50_ of 0.36 μM and oral administration of EGCG lowered the accumulation of 3-*O*-methyl-DOPA in the plasma and striatum of rats treated with DOPA plus carbidopa, the most commonly used treatment for symptom management in PD. EGCG also reduced glutamate-induced oxidative cytotoxicity in cultured HT22 mouse hippocampal neuronal cells by inactivating the nuclear factor-κB-signaling pathway.

The antioxidant and metal chelation activities of EGCG and GTCs are important for the prevention of neurodegenerative diseases. Iron in the ferrous form, Fe(II), can react with hydrogen peroxide via the Fenton reaction, leading to the generation of hydroxyl radicals that are extremely reactive and damage cellular components [60]. Copper is able to form a high-affinity complex with Aβ, and the oxidation reactions by Cu(II)-Aβ complexes lead to the formation of Cu(I)-Aβ, which is involved in neurotoxicity via radical cation and hydroxyl radical generation [61]. EGCG and GTCs containing many hydroxyl groups in their structure can have both antioxidant property to terminate free radical chain reactions and play the role of chelator of redox-active metals [56]. EGCG has also been shown to act as a pro-oxidant in addition to the known antioxidant activity, directly regulate biological actions as a transcription factor, and act as a member in signal transduction pathway and as an inducer of DNA methylation [62].

Other studies examined the protective effects of EGCG on lipopolysaccharide (LPS)-mediated inflammation and neurotoxicity. Liu et al. treated macrophages with LPS to induce the expression of proinflammatory cytokines (TNF-α, IL-1β, and IL-6). They found that EGCG inhibited LPS-mediated induction of these cytokines and that a supernatant from EGCG-pretreated and LPS-activated macrophage cultures was less cytotoxic to neurons than from non-EGCG-pretreated and LPS-activated macrophage cultures. Moreover, EGCG inhibited the LPS-induced production of ROS in neurons. Thus, EGCG represents a potent and useful neuroprotective agent for inflammation-mediated neurological disorders [63].

Catuara-Solarz et al. explored the effects of a combined therapy with EE and EGCG in the Ts65Dn mouse model of DS in young mice. Their results showed that combined EE-EGCG treatment improved corticohippocampal-dependent learning and memory. Cognitive improvement was accompanied by a rescue of cornu ammonis 1 (CA1) dendritic spine density and normalization of the proportion of excitatory and inhibitory synaptic markers in CA1 and dentate gyrus [64].

Stress is a risk factor for developing mental disorders. Chronic stress may lead to the hyperactivation of the hypothalamic-pituitary-adrenal axis and a sustained rise in the levels of glucocorticoids such as corticosterone. Feng et al. examined the effects of EGCG on corticosterone-induced neuronal damage using PC12 cells [65]. The results demonstrated that exposure to high concentrations of corticosterone induced cytotoxicity and downregulated the Sonic hedgehog (Shh) pathway in PC12 cells, but that EGCG decreased corticosterone’s effect. The Shh pathway inhibitor, cyclopamine, reduced the EGCG-mediated neuroprotective effects and upregulated the Shh pathway, suggesting that EGCG protects neural cells from corticosterone’s neurotoxicity via activation of Shh signaling.

Similarly, Zhao et al. examined the possible involvement of PKCα and ERK1/2 signaling pathways in EGCG-mediated protection against restraint stress-induced neural injuries [66]. EGCG and GTPs improved the restraint stress-induced neuronal impairments in rats accompanied by a partial restoration of normal plasma glucocorticoid, dopamine, and serotonin levels. In the stressed animals, EGCG decreased the stress-induced decrease in PKCα and ERK1/2 expression and phosphorylation, and restored the production of ATP and the expression of a key regulator of cellular energy metabolism, peroxisome proliferators-activated receptor-γ coactivator-1α. The finding suggested that EGCG might have protective effects on stress-induced neural injuries leading to neuropsychiatric disorders and psychosomatic diseases.

**Figure 2:**
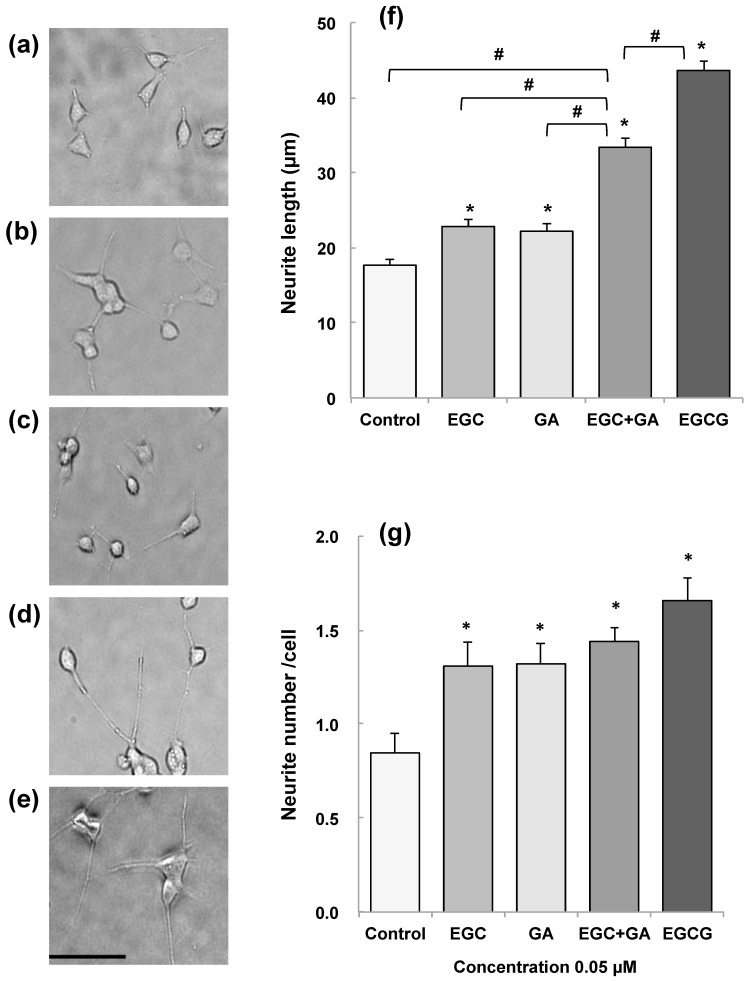
Effect of EGCG, EGC, and gallic acid (GA) on neurite outgrowth of human neuroblastoma SH-SY5Y cells [62]. EGCG, EGC, and GA dissolved in 0.01% dimethylsulfoxide were added to the culture medium to make a final concentration of 0.05 µM and cultured for 72 h at 37 °C. Photos of (**a**) control cells and (**b**) cells treated with EGC, (**c**) GA, (**d**) EGC and GA, and (**e**) EGCG. (**f**) Neurite length and (**g**) neurite number of cells treated with catechins. Scale bar is 50 µm. Each value represents the mean ± SEM. Asterisks and # represent significant differences with the control (*) and with EGC and GA (#) (*p* < 0.05, Bonferroni’s *t*-test). Reproduced under Creative Commons Attribution-Noncommercial-No Derivatives License (CC BY NC ND). doi:10.1016/j.bbrep.2016.12.012.

During the course of studies to examine whether metabolites of EGCG may have a neuroprotective effect comparable to EGCG, in vitro blood–brain barrier (BBB) permeability after exposure to 30 min of EGCG, EGC, and GA was found to be about 2.8, 3.4, and 6.5% of the initial permeability, respectively [67]. The permeability of EGCG indicates that EGCG reached the brain parenchyma even at very low concentrations. The learning ability of senescence-accelerated animal model SAMP10 mice that ingested EGCG (20 mg/kg) was significantly higher than that of mice that ingested either EGC or GA alone. Combined ingestion of EGC and GA resulted in a significant improvement that was comparable to the effect of EGCG. SH-SY5Y cell growth was significantly enhanced by 0.05 µM EGCG, but this effect diminished at higher concentrations, indicating that an optimal concentration of EGCG is required in cell proliferation. The length and number of neurites were significantly higher in cells treated with catechins than in control cells (Figure 2). Neurite length was significantly greater in cells treated with EGCG than in untreated cells (Figure 2). The combination of EGC and GA more efficiently induced neurite outgrowth than either EGC or GA alone (Figure 2). These results indicate that EGCG and the combination of EGCG’s hydrolysis products EGC and GA can suppress cognitive dysfunction after passing the BBB and that EGCG and the combination of EGC and GA induce neurite outgrowth efficiently, suggesting that oral administration of EGCG can exert a protective effect on brain function even when it undergoes in vivo hydrolysis to EGC and GA [67].

## 5. Computational Molecular Docking Analysis (CMDA)

The development of CMDA has contributed to an understanding of the molecular basis of the mechanism by which GTCs exert beneficial effects on neurodegenerative diseases. In a comprehensive review article, Lemkul and Bevan discussed how techniques, such as CMDA and molecular dynamics simulations, can be applied in developing small molecules as therapeutics for preventing the pathogenic aggregation of Aβ, which is one of the hallmarks of the progression of AD. These techniques showed that EGCG binds to Aβ(1–42)(Aβ42), revealing the 12 amino acid residues to which EGCG principally binds. Polar interactions, such as hydrogen bonding, play only a minor role in this process, and an excess concentration of EGCG efficiently prevented the increase in β-strand content in the polypeptide leading to inhibited aggregation. As described above, EGCG inhibits the fibrillogenesis of Aβ42 and reduces its associated cytotoxicity [68]. The exclusion of water from the surface of Aβ and the resulting interactions between EGCG and Aβ may be responsible for the structural change in Aβ. Thus, the affinity of EGCG for many residues in the Aβ amino acid sequence could explain its strong inhibitory effect on aggregation [69].

Using isothermal titration calorimetry and CMDA, Wang et al. examined the interactions between Aβ42 and EGCG at different conditions including multiple temperatures and EGCG and Aβ42 concentrations. They found that binding stoichiometry was linearly related to the EGCG/Aβ42 molar ratio. The predominant interaction gradually shifted from hydrogen bonding to a hydrophobic interaction with an increase in this ratio, resulting in a transition of binding from an enthalpy-driven to an entropy-driven state. The binding of EGCG to Aβ42 was promoted by increasing temperature and salt concentration. Those findings have been useful for research on the inhibition of Aβ aggregation and new drug development [70].

HSP90α is involved in regulating the function of tau proteins that are involved in causing AD. Inhibition of HSP90α by *C*-Terminal domain ATP binding-site blockage may be an effective treatment strategy against the disease by degrading tau proteins. Khalid and Paul performed CMDA and showed that Leu665, Leu666, and Leu694 at this binding site might be the binding sites of HSP90α and HSP90 inhibitors, such as EGCG. The best binding energy was lower for Leu666 (−7.53 kcal/mol) than for Leu665 (−7.20 kcal/mol) and Leu694 (−6.67 kcal/mol). Based on a report that showed EGCG enhances the clearance of phosphorylated tau protein in primary neurons, EGCG may be considered as a promising therapeutic agent in the treatment of AD [71].

Similarly, using molecular dynamics simulations and molecular mechanics-Poisson Boltzmann surface area analysis, Liu et al. found that EGCG molecules expel water from the surface of Aβ42, cluster with each other, and interact directly with the polypeptide [72]. These authors identified 12 important residues of Aβ42 that strongly interact with EGCG (Phe4, Arg5, Phe19, Phe20, Glu22, Lys28, Gly29, Leu34-Gly37, and Ile41). Nonpolar interactions were mainly found between the side chains of some hydrophobic residues (Phe, Met, and Ile) and the main chains of some non-hydrophobic residues (Lys28 and Gly29). Polar interactions were mainly found between the main chain of Aβ42, in which the peptide bonds of Gly29 and Gly37 predominantly contributed because they have no side chain to cause steric hindrance. This study indicated that nonpolar interactions and hydrogen bonds are coupled to prevent the conformational conversion of Aβ42 and its following aggregation, and these findings appear to be critically important for exploring more effective agents for the inhibition of Aβ42 fibrillogenesis [72].

Ehrnhoefer et al. demonstrated that EGCG inhibits the fibrillogenesis of both α-synuclein and Aβ by directly binding to natively unfolded polypeptides and preventing their conversion into toxic aggregation intermediates. CMDA showed that EGCG preferentially bound the C-terminus of α-synuclein (Asp119, Ser129, Glu130, and Asp135). EGCG promoted the formation of unstructured, nontoxic α-synuclein and Aβ oligomers of a new type, instead of β-sheet-rich amyloid, suggesting its favorable effect on aggregation pathways in neurodegenerative diseases [73].

The Aβ oligomers, which are the main culprits in the cytotoxicity of AD and p3 peptides (Aβ17–42 fragments), are present in AD amyloid plaques. Chebaro et al. determined the structure of Aβ17–42 trimers both in aqueous solution and in the presence of five inhibitors such as EGCG, which can slow Aβ aggregation and reduce toxicity. Analyses including CMDA revealed that the conformational ensemble of the Aβ17–42 trimer can be described by 14 clusters with each peptide essentially adopting turn/random coil configurations, although the most populated cluster is characterized by one peptide with a β-hairpin at Phe19–Leu31. CMDA revealed that EGCG and other inhibitors have multiple binding modes with different binding affinities for trimeric Aβ17–42, by preferentially interacting with the region of the amino acid residues 17–21 [74].

**Figure 3:**
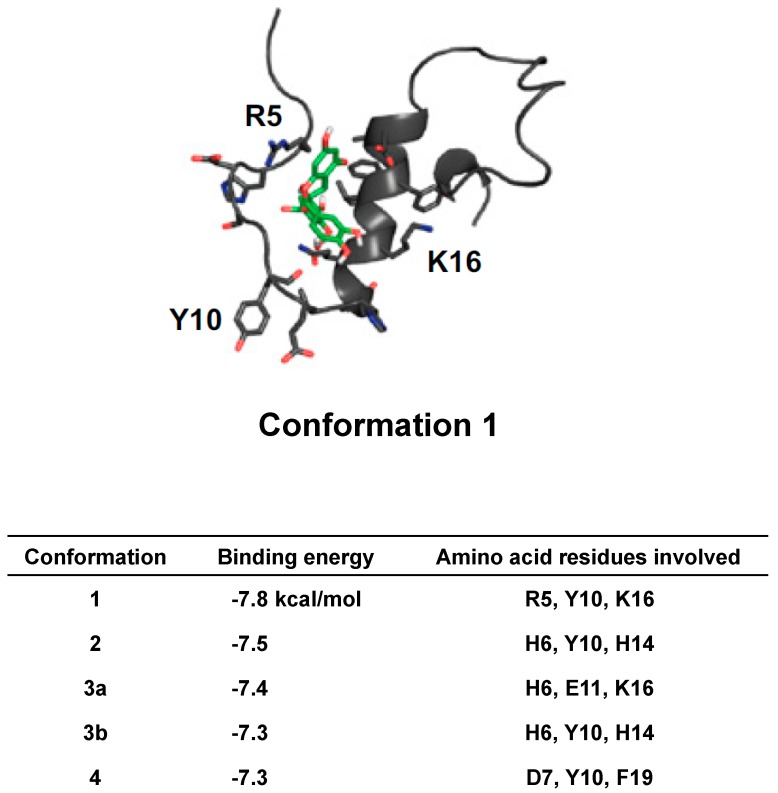
Computational molecular docking analysis (CMDA) of the EGCG and Aβ complex [70]. The possible pose conformation of the EGCG-Aβ complex with the highest frequency is shown with the involvement of Arg5, Tyr10, and Lys16. Binding energy and the amino acid residues involved in the top five pose conformations with a high frequency are also shown. Reproduced in part with permission of the publisher, Proceedings of the National Academy of Sciences of the United States of America. doi:10.1073/pnas.12203261.

Hyung et al. found that EGCG interacted with metal Aβ to form small, unstructured Aβ aggregates more distinctively than in Aβ. EGCG reduced the toxicity of both metal Aβ and metal-free Aβ in cultured neuroblastoma cells. The results, including that of CMDA, showed that EGCG bound to Aβ monomers and dimers to form more compact peptide conformations than those from EGCG-untreated Aβ species and that ternary EGCG-Aβ complexes were produced. Figure 3 shows the possible conformation of the EGCG-Aβ complex with the lowest binding energy of −7.8 Kcal/mol (Conformation 1) together with the amino acid residues involved in the top five conformations with high frequency. The interactions of EGCG with Arg5, Tyr10, and Lys16 were observed in Conformation 1 with eight amino acid residues, Arg5, His6, Asp7, Tyr10, Glu11, His14, Lys16, and Phe19. Phe19 was involved in the top five pose conformations and was also found in the amino acid residues of the top 10 pose conformations, suggesting that these residues are quite important in the interaction between EGCG and Aβ. These findings demonstrate the anti-amyloidogenic activity of EGCG toward metal Aβ species with a structure-based mechanism (Figure 3) [75].

The protein ataxin-3 (ATX3) triggers an amyloid-related neurodegenerative disease when its polyglutamine stretch is expanded beyond a critical threshold. Previously, EGCG was shown to redirect amyloid aggregation of a full-length, expanded ATX3 (ATX3-Q55) toward non-toxic, soluble, sodium dodecylsulfate-resistant aggregates. Visentin et al. found a similar action of EGCG, EGC, and GA [76]. These three polyphenols prevented the appearance of ordered side-chain hydrogen bonding in ATX3-Q55, which is the hallmark of polyglutamic acid-related amyloids. CMDA showed that all three compounds bound to each of the three interacting regions with different patterns, including the central aggregation-prone region, which might account for their ability to prevent amyloidogenesis. These three compounds reduced ATX3-Q55′s cytotoxicity in neural cells and in a transgenic *Caenorhabditis elegans* strain expressing expanded ATX3. Although EGCG, EGC, and GA act in a similar manner, GA might be more suitable for antiamyloid treatments due to its simpler structure and higher chemical stability.

## 6. Conclusions

Several epidemiological and human intervention studies have found beneficial effects of the consumption of tea and green tea on neurodegenerative impairment, such as cognitive dysfunction and memory loss. In more specific brain disorders, studies have supported the beneficial effects of tea on PD, but few studies have revealed such effects on AD. However, several human studies have failed to show tea’s favorable effects on neurodegenerative diseases. This discrepancy may be due to several confounding factors, including the method used to quantify consumption, beverage temperature, cigarette smoking, alcohol consumption, and differences in genetic and environmental factors such as race, sex, age, and lifestyle [1,2,77]. Intestinal microbiota and genetic polymorphisms may also have influenced the results [2,78]. Therefore, more rigorous human studies are required to understand teas’ neuroprotective effects. The results of a number of laboratory experiments have demonstrated the benefits of green tea and EGCG and proposed mechanisms of action. The targets of GTCs include the abnormal accumulation of fibrous proteins such as Aβ and α-synuclein, inflammation, elevated expression of pro-apoptotic proteins, and oxidative stress, which are associated with neuronal cell dysfunction and death in the cerebral cortex. CMDA revealed how EGCG can prevent the accumulation of fibrous proteins. These findings suggest that GTCs have the potential to be used in the prevention and treatment of neurodegenerative diseases and should be useful for the development of new drugs.

## Figures and Tables

**Table 1:** Examples of observational epidemiological studies on the effect of tea consumption on cognitive decline or impairment.

	Primary Author (Year)	Favorable Effect	Reference
1	Kuriyama (2006)	Yes	[20]
2	Ng (2008)	Yes	[21]
3	Huang (2009)	Yes	[22]
4	Feng (2010)	Yes	[23]
5	Noguchi-Shinohara (2014)	Yes	[25]
6	Mashal (2013)	No	[28]
7	Nurk (2009)	Yes	[29]
8	Corley (2010)	No	[30]
9	Wu (2011)	No	[31]
10	Feng (2012)	Yes	[32]
11	Wang (2014)	No	[33]
